# Effect of changes in monitor unit rate and energy on dose rate of total marrow irradiation based on Linac volumetric arc therapy

**DOI:** 10.1186/s13014-019-1296-y

**Published:** 2019-05-27

**Authors:** Jaeman Son, Noorie Choi, Jung-in Kim, Jong Min Park, Hong-Gyun Wu, Hyun-Cheol Kang, Chang Heon Choi

**Affiliations:** 1 Department of Radiation Oncology, Seoul National University Hospital, Seoul, Republic of Korea; 2 Department of Radiation Oncology, Veterans Health Service Medical Center, Seoul, South Korea; 3 Department of Radiation Oncology, Seoul National University College of Medicine, Seoul, South Korea; 4 Biomedical Research Institute, Seoul National University Hospital, Seoul, Republic of Korea; 5 Institute of Radiation Medicine, Seoul National University Medical Research Center, Seoul, Republic of Korea; 6 Center for Convergence Research on Robotics, Advanced Institutes of Convergence Technology, Suwon, Republic of Korea

**Keywords:** Total marrow irradiation, Dose rate, Pulmonary toxicity, Volumetric arc therapy, Monitor unit rate

## Abstract

**Background:**

This study set out to evaluate the effect of dose rate on normal tissues (the lung, in particular) and the variation in the treatment efficiency as determined by the monitor unit (MU) and energy applied in Linac-based volumetric arc therapy (VMAT) total marrow irradiation (TMI).

**Methods:**

Linac-based VMAT plans were generated for the TMI for six patients. The planning target volume (PTV) was divided into six sub-volumes, each of which had their own isocenter. To examine the effect of the dose rate and energy, a range of MU rates (40, 60, 80, 100, 300, and 600 MU/min) were selected for 6, 10, and 15 MV. All the plans were verified by portal dosimetry.

**Results:**

The dosimetric parameters for the target and normal tissue were consistent in terms of the energy and MU rate. The beam-on time was changed from 59.6 to 6 min for 40 and 600 MU/min. When 40 MU/min was set for the lung, the dose rate delivered to the lung was less than 6 cGy/min (that is, 90%), while the beam-on time was approximately 10 min. The percentage volume of the lung receiving 20 cGy/min was 1.47, 3.94, and 6.22% at 6, 10, and 15 MV, respectively. However, for 600 MU/min, the total lung volume received over 6 cGy/min regardless of the energy, and over 20 cGy/min for 10 and 15 MV (i.e., 54.4% for 6 MV).

**Conclusions:**

In TMI treatment, reducing the dose rate administered to the lung can decrease the incidence of pulmonary toxicity. To reduce the probability of normal tissue complications, the selection of the lowest MU rate is recommended for fields including the lung. To minimize the total treatment time, the maximum MU rate can be applied to other fields.

**Electronic supplementary material:**

The online version of this article (10.1186/s13014-019-1296-y) contains supplementary material, which is available to authorized users.

## Background

Total body irradiation (TBI) has long played an important role as a conditioning regimen prior to bone marrow or hematopoietic stem cell transplantation for patients with hematologic malignancies (i.e., leukemia, lymphoma, or multiple myeloma) [1]. The primary reason for the use of TBI is the elimination of residual cancer cells and preventing the immunologic rejection of the transplanted donor stem cells [2]. The TBI treatments are generally performed at an extended source-to-surface distance (SSD) with large open fields to deliver a homogeneous dose to the entire body [3, 4]. In addition, TBI treatments have been performed using techniques such as moving-beam TBI with a sweeping beam, a patient translation technique, multiple-beam TBI, a shrinking-field technique, and the local application of small beams [5–7].

However, for patients with advanced acute leukemia, increasing the total TBI dose may improve malignant clone killing, but it is also associated with a potential lethal toxicity to the surrounding normal healthy tissues/organs [8, 9]. The side effects of TBI can be extensive and include acute symptoms such as nausea and a loss of appetite, as well as infertility and secondary malignancies [10]. Pulmonary toxicity is one of the most serious, and potentially life-threatening, complications that may occur following TBI [11]. Therefore, new techniques have concentrated on providing a safer dose escalation [12, 13].

To deliver a therapeutic dose to a target, while not irradiating normal tissue, total marrow irradiation (TMI) has been introduced in combination with helical tomotherapy (HT) or volumetric arc therapy (VMAT) using conventional linear accelerators [12, 14, 15]. TMI, when delivered with an intensity-modulation (IM) technique, may reduce the dose delivered to the organs at risk (OARs), relative to TBI. These techniques have the potential to provide a safer dose escalation for a target [16]. Several studies have shown the potential clinical benefits of IM-TMI [12–15, 17, 18].

For conventional TBI treatments, the dose rate is a significant parameter. To minimize the dose rate, the patient is generally located at an extended SSD of more than 400 cm [3]. Many studies have reported that, when TBI is performed with a low dose rate, the healthy tissue exhibits less damage [8, 9, 11, 19, 20]. Especially, low dose rates (less than 6 cGy/min) can decrease the risk of pulmonary toxicity [11, 19, 21]. However, TMI treatments with HT or VMAT have conventionally been performed with relatively high dose rates due to limitations on the dose rate selection and the treatment location (i.e., at the isocenter). For example, the lowest dose rate is 100 monitor units (MU)/min and 800 MU/min for the Varian C-series Clinac® (Varian Medical Systems, Palo Alto, CA) and HT, respectively. New delivery platforms that can deliver low dose rates have been released by Varian. Their VitalBeam™ (Varian Medical Systems, Palo Alto, CA) and Truebeam™ systems (Varian Medical Systems, Palo Alto, CA) offer dose rates of as low as 5 MU/min and 20 MU/min for 6–10 MV and 15–20 MV, respectively. In VMAT delivery mode, the dose rate can be varied during the treatment. However, the maximum dose rate can be limited by setting the dose rate in the planning process. If, in the plan optimization, the dose rate is set to less than 100 MU/min, which is supported by the C-series, a safe dose rate can be delivered to the OARs, especially the lungs.

In this paper, we describe the plan quality of TMI treatments with a range of MU rates and energies, performed using a Vitalbeam system. The appropriate MU rate setting was determined by evaluating the dose distribution, the dose rate to each OAR, and the duration of the TMI treatment.

## Materials and methods

### Patient selection and simulation

Six patients being treated by TMI were enrolled in the present study. Their participation in the study was approved by the institutional review board (IRB No. H-1706-007-855). The height and weight of each patient are listed in Table 1. All the patients were scanned using the Brilliance CT Big Bore scanner (Philips, Amsterdam, Netherlands) with a 3.0-mm slice thickness.

**Table 1:** Patient information (height, weight, and gender)

Patient	Height (cm)	Weight (kg)	Gender
1	185.6	72.2	M
2	161.5	51.3	F
3	170	73.1	M
4	163.2	62.5	F
5	163.6	64.8	M
6	164.8	55.9	M

### Treatment plan

A radiation oncologist defined the clinical target volume (CTV) including all the bone marrow in the body from the head to the mid-thighs, but excluding the forearms and hands. The CTVs were segmented for the bone, brain, liver, spleen, and testis. Regarding the lower extremities, one third of the upper femur (active marrow region) was included for CTV definition.

To define the planning target volume (PTV), each CTV was extended by adding a different margin in consideration of the internal motion of each organ. The brain, bone and testis PTV was defined by adding 3, 5, and 7 mm to their CTVs, respectively. For the liver and spleen PTV, a 5-mm margin was set in all except the superior-inferior (SI) directions. For the SI direction, a 15-mm margin was applied.

The total PTV was defined as the sum of each PTV. The total PTV was divided into sub PTVs for each isocenter. A total of six isocenters were defined considering the shape and length of the PTV. The sub PTVs were named as follows: PTV_Brain, PTV_Chest1, PTV_Chest2, PTV_Abdomen, PTV_Pelvic, and PTV_Femur. The lungs, kidneys, eyes, oral cavities, bowels, and heart were designated as being OARs. The prescription dose was 10 Gy, administered in five fractions [12]. The dose constraints of the mean doses administered to the lungs, kidneys, bladder, and bowels were set to 6, 7, 8.5, and 7 Gy, respectively. All the plans were normalized to cover 90% of the PTV volume with 100% of the prescription dose. The patient was treated once a day with a 2-Gy dose, administered by the Vitalbeam system.

A VMAT-TMI treatment plan was generated using an Eclipse™ system (Ver. 13.7, Varian Medical Systems, Palo Alto, CA) with PRO 13.7 (progressive resolution optimization, Ver. 13.7, VarianTM Medical Systems, Palo Alto, CA) as the optimizer algorithm. The dose calculation was performed using Acuros® XB 13.7 with a 2.5-mm grid.

**Fig. 1:**
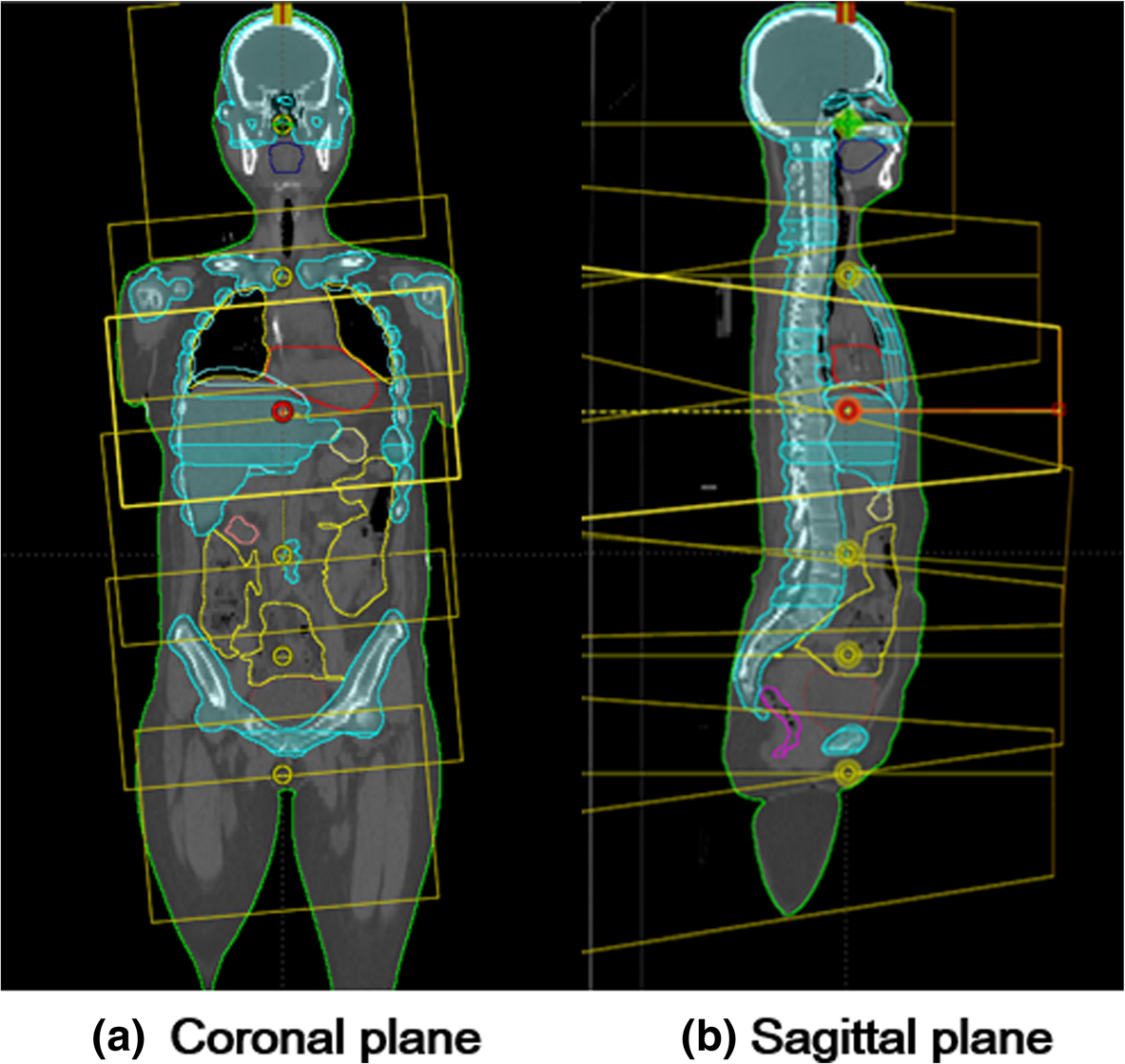
Field arrangement for VMAT. Pictorial representation of total marrow irradiation (TMI) plan by volumetric arc therapy (VMAT) consisting of six isocenters with a single arc. Field arrangement projected at isocenter in coronal plan (**a**) and sagittal plane (**b**)

A total of six arc fields corresponding to each isocenter were created with inter-gantry angles of 179° and 181°. Each field was fully opened to cover the entire length of each sub PTV with a collimator rotation of 90° or 180°. For TMI using a multi-isocenter, the verification of the dose accumulation in a junction area should be considered. Previous studies investigated the robustness of the junctions [15, 17, 18]. Those studied have suggested at least 2 cm overlapped region between the previous and following arc on each side to eliminate any hot and cold spots. In the present study, the overlapping regions between neighboring fields were set with at least 2 cm on each side to avoid there being hot or cold regions around the field junctions following literature [15, 17, 18]. Figure 1 shows the arrangement of the field and isocenter corresponding to each PTV.

First, a VMAT-TMI plan was generated by changing the energy (6, 10, and 15 MV) with a dose rate of 600 MU/min, while maintaining the collimator angles, field sizes, and number and location of the isocenters for the total field. Next, new plans were created in accordance with the changing MU rate (40, 60, 80, 100 and 300 MU/min) for each energy. For new created plans, the each VMAT optimization was performed with same optimization parameters.

One MU was calibrated while 1 cGy was delivered to the maximum depth (e.g., 1.5, 2.5, and 2.9 cm depth for 6, 10, and 15 MV) in a water phantom with a 100-cm source to surface distance and a 10 × 10 cm^2^ field size.

### Plan analysis

The beam-on time was calculated to evaluate the dose rate for each field. To evaluate the non-linear correlation between the dose rate and beam-on time, the non-linear correlation factor was defined as the ratio of MUs to beam-on times. The dosimetric quality was evaluated with respect to the target coverage and OAR dose. For the PTV, the dose parameters were analyzed using the near-minimum (D_98%_), near-maximum, (D_2%_), and mean absorbed doses, the latter being designated D_50%_. For the OAR dose, the mean dose and V_5Gy_ (cc) were evaluated.

The average dose rate delivered to the lungs was calculated by dividing the mean dose by the beam-on time. According to the guidelines published by the ACR and the ASTRO, a dose rate to the lungs of less than 20 cGy/min has been suggested to decrease the incidence of pulmonary toxicity. Therefore, the average volume irradiated at a dose rate of more than 20 cGy/min was evaluated for the lungs. The PTV_Chest1 and PTV_Chest2 isocenters were set to irradiate the chest region including the lungs and ribs. The sum of the beam-on time for PTV_Chest1 and PTV_Chest2 was used as the beam-on time for calculating the average dose rate for the entire lung.

### Plan verification

The plans were verified using a Portal dosimetry system with an electronic portal imaging device. The verification plans were created using a portal dose image prediction (PDIP, Ver 13.7, Varian Medical Systems, Inc., Palo Alto, CA) algorithm. For gamma index evaluation, the criteria was set to a 2% dose difference and a 2-mm distance to agreement. The threshold dose was set to 10%.

## Results

**Fig. 2:**
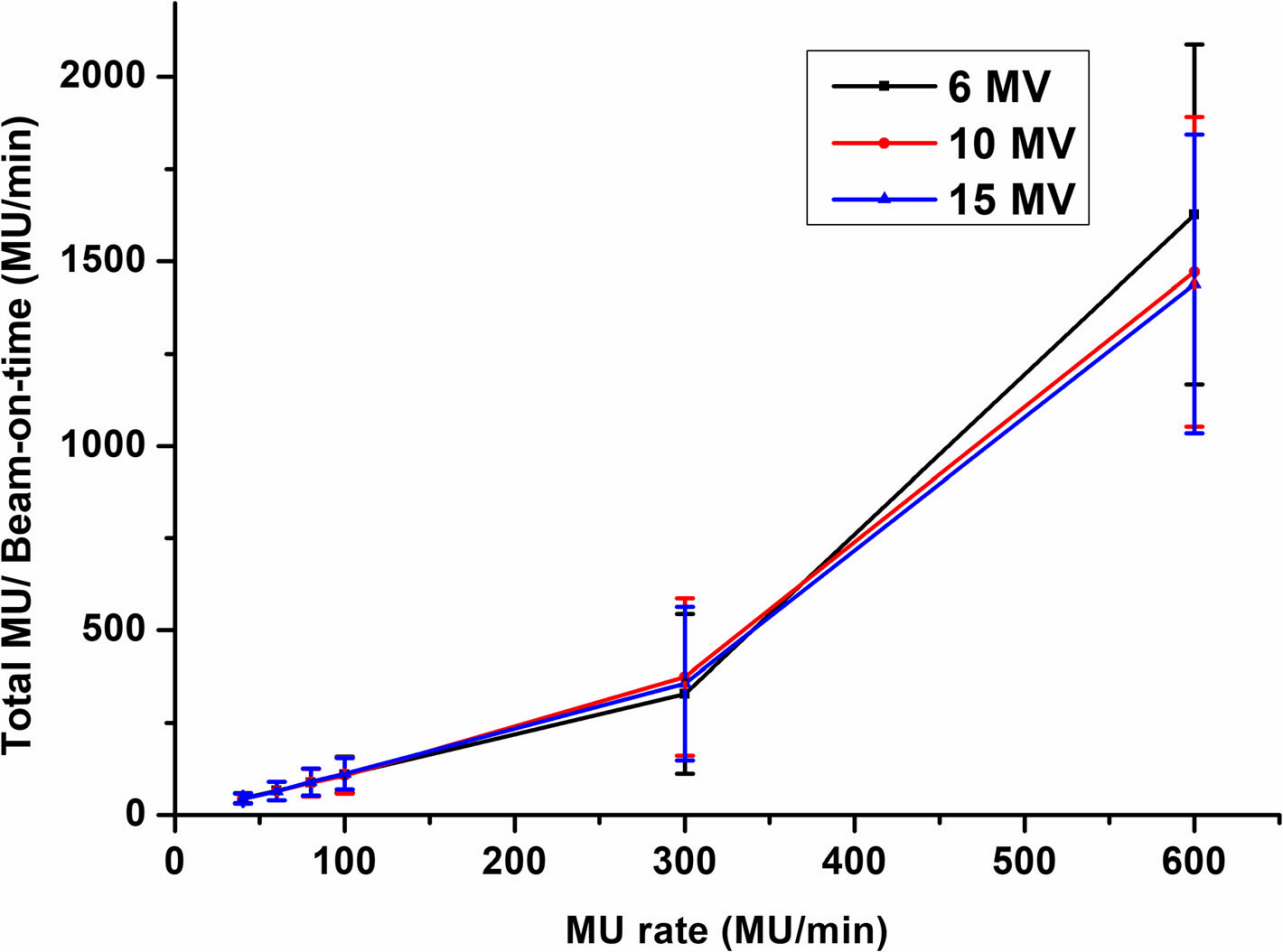
Non-linear correlation factor. The factors were defined as a ratio of total MU to beam-on time. 6 MV (black solid line), 10 MV (red solid line) and 15 MV (blue solid line)

The beam-on times and MU for each field, energy, and dose rate are listed in Table 2. Figure 2 shows the non-linear correlation factor between the MUs and beam-on times. The beam-on time decreases as the energy increases. The beam-on time for field 1 (PTV_Brain) was the shortest. The non-linear correlation factor increases dramatically with the MU rate.

**Table 2:** Beam-on time and MU of each isocenter for energy and MU rate

Energy (MV)	MU rate (MU/min)	Isocenter						Total
		PTV						
		Brain	Chest1	Chest2	Abdomen	Pelvic	Femur	
		Beam-on time (min)						
		Monitor Unit (MU)						
6	40	8.6 ± 2.7	11.6 ± 1.9	10.5 ± 2.5	10.6 ± 2.6	11.2 ± 2.7	11.4 ± 3.7	63.9 ± 14.5
		382.3 ± 109.6	483.8 ± 64.1	458.7 ± 86.2	455.8 ± 90.6	493.2 ± 97.1	524.3 ± 166.9	2798.1 ± 523.9
	60	5.8 ± 1.8	7.9 ± 1.3	7.1 ± 1.7	7.1 ± 1.8	7.6 ± 1.8	7.7 ± 2.5	43.1 ± 9.7
		372.2 ± 96.7	488.2 ± 66.2	464.5 ± 82.5	461.8 ± 89.2	499.5 ± 94.4	529.5 ± 161.9	2815.7 ± 505.8
	80	4.4 ± 1.5	5.9 ± 1.0	5.3 ± 1.3	5.4 ± 1.4	5.7 ± 1.4	5.8 ± 1.9	32.5 ± 7.8
		373.3 ± 108	492.8 ± 66.7	467.5 ± 90.2	464.2 ± 95.7	501.8 ± 97.6	533 ± 165.9	2832.6 ± 547.6
	100	3.5 ± 1.1	4.7 ± 0.8	4.2 ± 1.0	4.3 ± 1.1	4.5 ± 1.1	4.6 ± 1.5	25.8 ± 5.8
		373.8 ± 97.9	491.3 ± 64.4	463.8 ± 83.5	464.4 ± 89.4	499.2 ± 94.9	529.3 ± 163.5	2821.8 ± 512
	300	1.3 ± 0.3	1.6 ± 0.3	1.4 ± 0.3	1.5 ± 0.3	1.6 ± 0.3	1.6 ± 0.5	8.9 ± 1.8
		370.3 ± 97.9	489.8 ± 65.2	464.2 ± 83.7	462.3 ± 89.4	498.7 ± 94.5	529.8 ± 164.2	2815.1 ± 511.6
	600	1.0 ± 0.0	1.0 ± 0.0	1.0 ± 0.0	1.0 ± 0.0	1.0 ± 0.1	1.1 ± 0.1	6.1 ± 0.3
		371 ± 98	490.8 ± 64.5	465.2 ± 84.5	461.7 ± 89.6	492.3 ± 88.8	528.7 ± 162.7	2809.7 ± 508.7
10	40	8.0 ± 2.5	10.6 ± 1.8	9.6 ± 2.3	9.7 ± 2.5	10.4 ± 2.5	10.5 ± 3.3	58.8 ± 13.6
		339.8 ± 93	441.5 ± 59.8	420.8 ± 77.3	419.8 ± 83.5	459.7 ± 92.4	478.2 ± 144.4	2559.8 ± 476.9
	60	5.3 ± 1.7	7.1 ± 1.2	6.4 ± 1.6	6.5 ± 1.6	6.9 ± 1.6	7.0 ± 2.3	39.2 ± 9.1
		338.4 ± 92.6	445.4 ± 59.2	423.3 ± 78.3	420 ± 83.6	455.8 ± 87.3	482.3 ± 148.3	2565.2 ± 479.4
	80	4.0 ± 1.3	5.4 ± 0.9	4.8 ± 1.2	4.9 ± 1.2	5.2 ± 1.2	5.3 ± 1.7	29.4 ± 6.8
		338.2 ± 92.4	446.8 ± 59.6	424.4 ± 78.2	421.2 ± 83.7	455.3 ± 86.3	483.8 ± 148.9	2569.7 ± 479.6
	100	3.2 ± 1.0	4.3 ± 0.7	3.9 ± 0.9	3.9 ± 1.0	4.1 ± 1.0	4.2 ± 1.4	23.6 ± 5.4
		337.4 ± 92.3	446.2 ± 59.4	423.5 ± 79	423.1 ± 83.4	454.8 ± 88.2	482.5 ± 149.3	2567.5 ± 480.1
	300	1.2 ± 0.2	1.4 ± 0.2	1.3 ± 0.3	1.4 ± 0.3	1.4 ± 0.3	1.5 ± 0.4	8.2 ± 1.5
		337.3 ± 92.5	446.8 ± 59.2	422.8 ± 78.8	421.5 ± 83.7	454.9 ± 87.3	483.3 ± 149.6	2566.6 ± 479.6
	600	1.0 ± 0.0	1.0 ± 0.0	1.0 ± 0.0	1.0 ± 0.0	1.0 ± 0.1	1.0 ± 0.1	6.1 ± 0.2
		336.5 ± 92.6	446.3 ± 59.4	422.6 ± 78.9	420.2 ± 83.5	453.2 ± 87.7	482.3 ± 147.2	2561.1 ± 479.8
15	40	7.6 ± 2.4	10.2 ± 1.7	9.2 ± 2.2	9.3 ± 2.3	9.8 ± 2.3	10.0 ± 3.3	56.1 ± 12.9
		321.8 ± 89	424.8 ± 55.8	403.3 ± 75.8	399.8 ± 79.9	432.5 ± 83.4	459.5 ± 143.8	2441.7 ± 460.9
	60	5.0 ± 1.6	6.8 ± 1.1	6.1 ± 1.5	6.2 ± 1.5	6.5 ± 1.5	6.7 ± 2.2	37.3 ± 8.5
		323.3 ± 88.6	424.8 ± 54.8	402.3 ± 75.6	399.2 ± 78.1	433.2 ± 84.3	458.3 ± 142.1	2441.1 ± 453.8
	80	3.8 ± 1.2	5.1 ± 0.8	4.6 ± 1.1	4.6 ± 1.2	4.9 ± 1.1	5.0 ± 1.6	28.0 ± 6.4
		322.1 ± 88.2	422.1 ± 56.3	402.8 ± 75.1	397.2 ± 77.2	431.8 ± 85.2	459.3 ± 143.2	2435.3 ± 453.8
	100	3.0 ± 0.9	4.1 ± 0.7	3.7 ± 0.9	3.7 ± 0.9	3.9 ± 0.9	4.0 ± 1.3	22.4 ± 5.1
		321.2 ± 87.3	420.2 ± 56.1	401.7 ± 77.6	398.5 ± 79.5	431.2 ± 81.2	548.2 ± 144.5	2521 ± 453.8
	300	1.2 ± 0.2	1.4 ± 0.2	1.3 ± 0.2	1.3 ± 0.2	1.4 ± 0.3	1.4 ± 0.4	7.9 ± 1.4
		319.3 ± 88.6	425.3 ± 55.1	400.6 ± 76.1	399 ± 80.1	430.9 ± 83.3	457.3 ± 142.3	2432.4 ± 453.8
	600	1.0 ± 0.0	1.0 ± 0.0	1.0 ± 0.0	1.0 ± 0.0	1.0 ± 0.1	1.0 ± 0.1	6.0 ± 0.2
		320.5 ± 88.8	423.2 ± 55.9	401.1 ± 75.3	398.2 ± 77.2	431 ± 81.5	457.5 ± 143.9	2431.5 ± 454.2
*P* value	40	0.828	0.447	0.685	0.711	0.700	0.810	0.669
		0.612	0.305	0.545	0.583	0.585	0.789	0.515
	60	0.769	0.392	0.636	0.655	0.621	0.777	0.603
		0.695	0.232	0.459	0.497	0.477	0.750	0.456
	80	0.756	0.373	0.625	0.642	0.605	0.762	0.592
		0.683	0.215	0.449	0.490	0.462	0.732	0.447
	100	0.771	0.398	0.646	0.663	0.628	0.781	0.612
		0.695	0.233	0.471	0.508	0.482	0.752	0.465
	300	0.800	0.417	0.680	0.699	0.689	0.828	0.647
		0.695	0.25	0.467	0.508	0.486	0.752	0.465
	600	1.000	0.519	0.650	0.759	0.955	0.930	0.842
		0.690	0.226	0.458	0.501	0.540	0.756	0.471

Dose-volumetric parameters for each MU rate of TMI by VMAT using 6, 10 and 15 MV was provided in supplementary material (Additional file 1: Table S1).

**Fig. 3:**
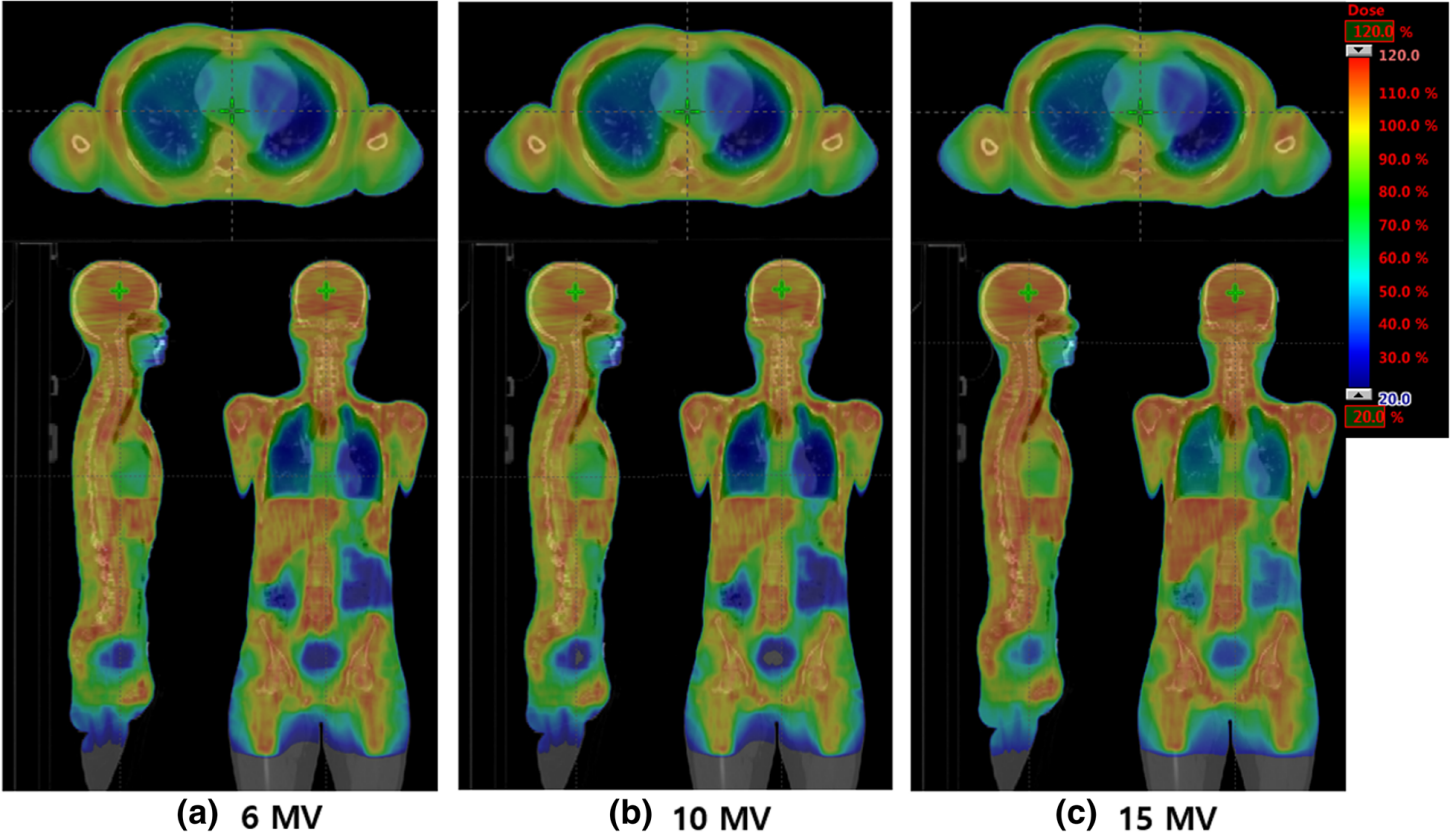
Isodose distributions. Isodose distributions in colorwash of TMI by VMAT for 6 (**a**), 10 (**b**), and 15 MV (**c**). Dose range: 20% (blue) to 120% (red)

The TMI isodose distributions in colorwash are illustrated in Fig. 3. The average values of the dose-volumetric parameters of PTV and normal tissue for the energy and dose rate of each patient are provided as supplementary material. The different dose-volumetric parameters for the energy and dose rates are not shown.

**Fig. 4:**
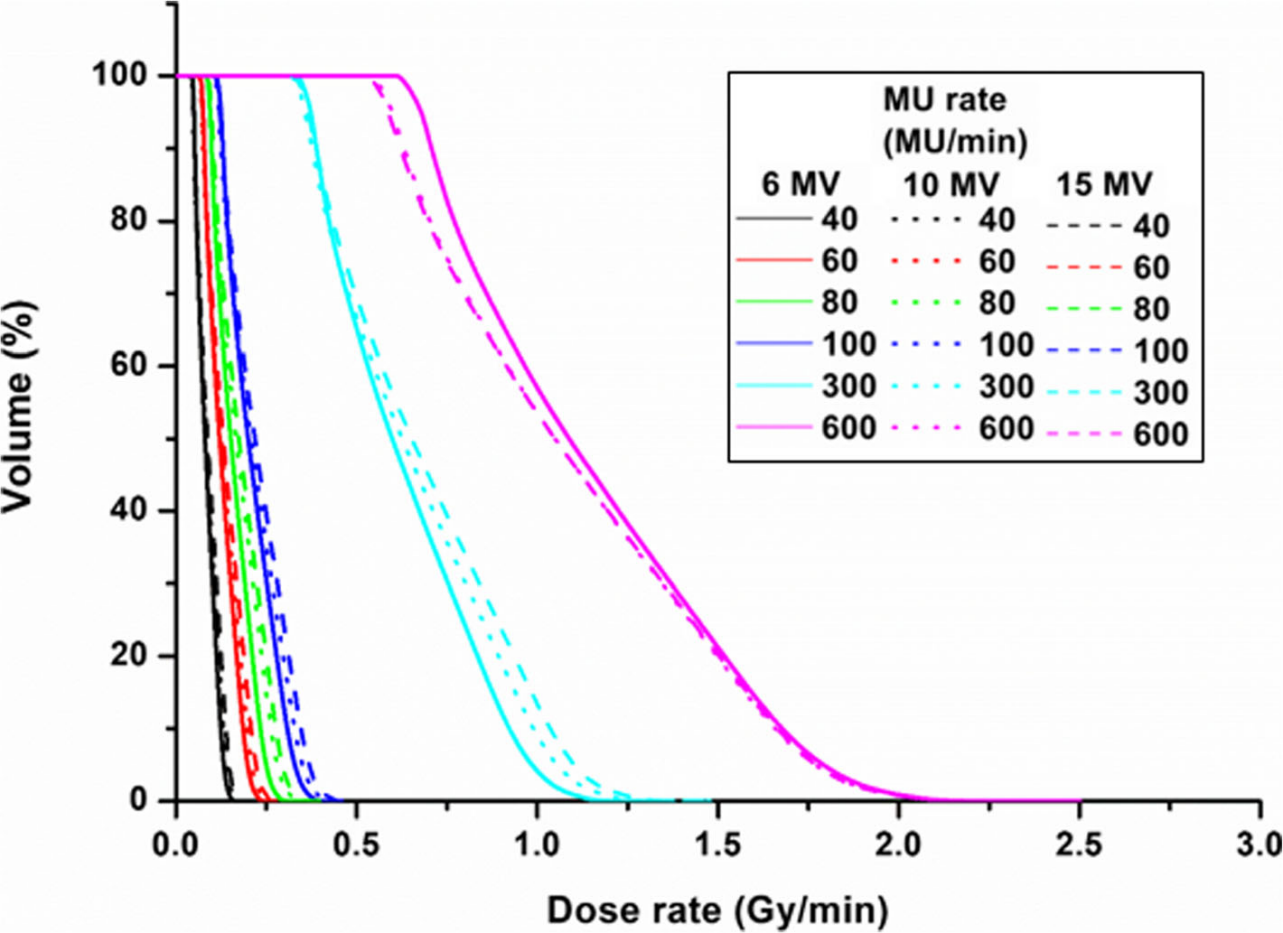
Dose-rate versus lung volume histogram. Dose-rate versus lung volume histogram of representative case for 6 MV (solid line), 10 MV (dotted line), and 15 MV (dashed line) according to the monitor unit (MU) rate. Black: 40 MU/min, red: 60 MU/min, green: 80 MU/min, blue: 100 MU/min, cyan: 300 MU/min and magenta: 600 MU/min

Figure 4 shows the dose-rate volumetric histogram for the lung volume of a representative case (Patient 1). When the 40-MU rate was used, the average volume of the lung receiving a dose of less than 6 cGy/min was 92.45 ± 8.88%, 93.47 ± 6.53% and 94.04 ± 6.11% at 6, 10, and 15 MV, respectively. The average lung volumes irradiated at a dose rate of more than 20 cGy/min are listed in Table 3.

**Table 3:** Average volume (%) of lung irradiated at dose rate of more than 20 cGy/min

Energy (MV)	MU rate (MU/min)					
	40	60	80	100	300	600
6	1.47 ± 1.56	24.42 ± 14.08	49.19 ± 14.28	69.59 ± 13.56	53.96 ± 28.69	54.40 ± 28.88
10	3.91 ± 3.90	30.81 ± 14.01	54.18 ± 12.02	71.24 ± 12.15	100.00 ± 0.00	100.00 ± 0.00
15	6.22 ± 6.14	35.10 ± 13.30	57.63 ± 11.59	74.58 ± 11.95	100.00 ± 0.00	100.00 ± 0.00

For all the plans, the gamma passing rate was more than 90%. For those plans with 300 and 600 MU/min at every energy, the gamma passing rate was 99%. For those plans with 40, 60, and 80 MU/min at every energy, the range of the gamma passing rate was 90–94%.

## Discussion

In the present study, we evaluated the dose rate and dosimetric parameter based on the MU rate of the machine and the energy.

TMI treatments using the VMAT technique are clinically favorable in that they can overcome the limitations of TBI since TMI can reduce the dose administered to an OAR. Additionally, the accuracy of the TMI treatment can be improved using an image guidance system. However, there remain issues with the dose rate when using TMI delivery with a C-series or HT device. However, this problem has been solved by the release of new machines with a low MU rate.

The radiation toxicity of a normal organ, especially the incidence of pulmonary toxicity, is a major factor determining the prescription dose for patients with hematolymphoid malignancies [8, 11, 19, 21]. Pulmonary toxicity can significantly affect the overall survival rate. The dose rate administered to the lung is a key factor, as well as the total dose. The published guidelines recommend that TBI treatments not exceed a dose rate of 20 cGy/min [21]. TBI with a dose rate of less than 6 cGy/min can reduce pulmonary toxicity [11, 19]. When 40 MU/min was set for the PTV_Chest1 and 2 fields, the lung volume receiving less than 6 cGy/min was 90% while the beam-on time of two field (PTV_Chest1 and 2) was 10 min, approximately. However, if MU/min was set for all the fields, the total treatment time would be 60 min. This long treatment time can result in increasing the intra-fraction error (e.g., breathing movement or patient motion. Moreover, the patient can feel discomfort due to lying down on the couch with an immobilization device. To avoid such an adverse effect, we can select the proper MU rate for a specific field which includes an OAR such as the lung. If the maximum MU rate (i.e., 600 MU/min) is applied to the PTV_Brain, Pelvic and Femur fields with 40MU/min for PTV_Chest1 and 2 only, the beam-on time was approximately 24 min. Even if the imaging acquisition time is included, the total treatment can be finished within 1 h. For imaging guide, cone beam computed tomography (CBCT) was taken for each single isocenter. The reference CT image set and digitally reconstructed radiograph was compare to CBCT and pair of orthogonal kV radiographs.

However, in terms of dose voxel, each voxel in OAR has a different dose rate. Each beam of field beam is subdivided into hundreds of beamlets. The voxel was partially irradiated by beamlet which have an individual intensity level [22]. In this study, the dose rate was calculated under the assumption that the dose to OAR is sum total of dose during beam on time.

The total dose administered to the lung did not exhibit any dependency on the energy. However, the beam-on time decreased slightly as the energy increased, except when the dose rate was 600 MU/min. A higher energy can result in the delivery of a similar dose in less time with a limited MU rate. However, when the maximum MU rate (600 MU/min) is allowed, the machine can deliver a sufficiently high dose rate at the maximum gantry speed. The beam-on time with an MU rate of 600 MU/min was constant since the beam-on time is predominantly determined by the maximum gantry speed.

The PTV should include the ribs since the bone marrow is primarily located in the ribs. The lungs, which are the most important OARs, are enclosed by the ribs. In planning the processing, we tried to reduce the dose administered to the lungs as far as possible. However, it was a difficult to reduce this dose due to the PTV coverage including the ribs. The DVH of the lung had a steep dose gradient with a range of 3–10 Gy.

The beam-on time and dose slightly decreased with an increase in the energy. With conventional TBI treatment, high-energy beams (10–18 MV) have historically been used, particularly for patients for which the thickness is greater than 35 cm, and therefore considered to be homogenous. However, when high-energy photon beams of more than 10 MV were used, the photoneutrons can lead to an increase in the undesired dose to the patient [23]. TMI techniques using 10 and 15 MV offer no advantages in consideration of the higher integral dose, larger penumbra and similar beam-on time and dose rate.

The variation in the MU rate was extremely limited relative to the general capacity of the machine. The optimization was processed with the extra constraints for a treatment plan with a lower dose rate. Therefore, plan verification was performed to check the delivery accuracy. The gamma passing rates of the plan with the lower MU rate were lower than those with the higher dose rate. The response of the Si detector used to perform portal dosimetry can be affected by the dose rate. The relatively low gamma passing rate was caused by this effect [24]. However, in every case, the gamma passing rate was more than 90%. All the plans could be delivered clinically.

In this study, the part of the lower extremity (i.e. lower legs from mid-femurs) was not included in VMAT optimization. The current treatment table on the LINAC has a maximum travel length of approximately 150 cm in longitudinal direction [25]. Therefore, two different plans are required for TMI which includes lower leg. In literature, reverse patient position approach has been introduced [18]. However, the field junction problem should be considered carefully for the approach [17]. VMAT plan could be generated for this part in feet-first position, keeping an overlapping region with the upper sector to prevent the risk of cold or hot spots [25].

## Conclusion

We evaluated the beam-on time and dose volume parameter for given energies and MU rates for VMAT TMI. The dose volume parameter was found to be not dependent on the energy and MU rate. However, the received PTV dose rate for normal tissue was found to differ significantly with the MU rate. In the case of TMI, the dose rate delivered to the lung is critical. To reduce the probability of normal tissue complications, the selection of the lowest MU rate was recommended for fields included the lung. Considering the total treatment time, maximum dose rate can be selected for other fields.

## Additional file

Additional file 1: **Table S1.** Dose-volumetric parameters for each MU rate of TMI by VMAT using 6, 10 and 15 MV. (XLSX 17 kb)

## Abbreviations

CTV: Clinical target volume
DVH: Dose volume histogram
IM: Intensity modulation
MU: Monitor unit
OAR: Organs at risk
PDIP: Portal dose image prediction
PTV: Planning target volume
TMI: Total marrow irradiation
VMAT: Volumetric modulated arc therapy
